# *In Vivo* Assay Reveals Microbial OleA Thiolases Initiating Hydrocarbon and β-Lactone Biosynthesis

**DOI:** 10.1128/mBio.00111-20

**Published:** 2020-03-10

**Authors:** Megan D. Smith, Serina L. Robinson, Mandkhai Molomjamts, Lawrence P. Wackett

**Affiliations:** a Biotechnology Institute, University of Minnesota, St. Paul, Minnesota, USA; b Department of Microbiology and Immunology, University of Minnesota, Minneapolis, Minnesota, USA; c Department of Biochemistry, Molecular Biology and Biophysics, University of Minnesota, Minneapolis, Minnesota, USA; d Microbial and Plant Genomics Institute, University of Minnesota, St. Paul, Minnesota, USA; University of Maryland, School of Medicine

**Keywords:** OleA, assay, bacteria, *para*-nitrophenol, screen, synthetic genes, thiolase

## Abstract

OleA, a member of the thiolase superfamily, is known to catalyze the Claisen condensation of long-chain acyl coenzyme A (acyl-CoA) substrates, initiating metabolic pathways in bacteria for the production of membrane lipids and β-lactone natural products. OleA homologs are found in diverse bacterial phyla, but to date, only one homodimeric OleA has been successfully purified to homogeneity and characterized *in vitro*. A major impediment for the identification of new OleA enzymes has been protein instability and time-consuming *in vitro* assays. Here, we developed a bioinformatic pipeline to identify OleA homologs and a new rapid assay to screen OleA enzyme activity *in vivo* and map their taxonomic diversity. The screen is based on the discovery that OleA displayed surprisingly high rates of *p*-nitrophenyl ester hydrolysis, an activity not shared by other thiolases, including FabH. The high rates allowed activity to be determined *in vitro* and with heterologously expressed OleA *in vivo* via the release of the yellow *p*-nitrophenol product. Seventy-four putative *oleA* genes identified in the genomes of diverse bacteria were heterologously expressed in Escherichia coli, and 25 showed activity with *p*-nitrophenyl esters. The OleA proteins tested were encoded in variable genomic contexts from seven different phyla and are predicted to function in distinct membrane lipid and β-lactone natural product metabolic pathways. This study highlights the diversity of unstudied OleA proteins and presents a rapid method for their identification and characterization.

## Abstract (precis)

Microbially produced β-lactones are found in antibiotic, antitumor, and antiobesity drugs. Long-chain olefinic membrane hydrocarbons have potential utility as fuels and specialty chemicals. The metabolic pathway to both end products share bacterial enzymes denoted as OleA, OleC, and OleD that transform acyl-CoA cellular intermediates into β-lactones. Bacteria producing membrane hydrocarbons via the Ole pathway additionally express a β-lactone decarboxylase, OleB. Both β-lactone and olefin biosynthesis pathways are initiated by OleA enzymes that define the overall structure of the final product. There is currently very limited information on OleA enzymes apart from the single representative from Xanthomonas campestris. In this study, bioinformatic analysis identified hundreds of new, putative OleA proteins, 74 proteins were screened via a rapid whole-cell method, leading to the identification of 25 stably expressed OleA proteins representing seven bacteria phyla.

## INTRODUCTION

**FIG 1:**
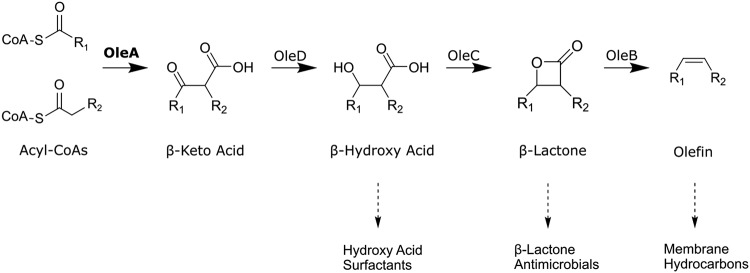
Bacterial metabolism using OleA proteins to initiate the pathways. Final products are from right to left, olefinic hydrocarbons that are components of membranes, β-lactones that serve as natural product enzyme inhibitors, and functionalized hydroxy acids that are produced by some bacteria as surfactants that act to solubilize hydrophobic substrates.

Bacteria produce different fatty acid derivatives that serve a structural role in cell membranes or that are secreted as natural products for intracellular signaling and competition (1–3). In most cases, those biosynthetic pathways are independent. Recently, a set of proteins denoted as OleA, OleC, and OleD showed overlapping function, being important in the microbial production of membrane hydrocarbons and β-lactone natural products (Fig. 1) (4).

Previously, we studied Ole enzymes in the model pathway found in the plant-pathogenic bacterium Xanthomonas campestris. In the X. campestris pathway, the OleA, OleC, and OleD enzymes are coexpressed with OleB, which catalyzes an unprecedented enzymatic decarboxylation of β-lactones (5) to generate hydrophobic membrane olefins (Fig. 1). However, other bacteria harbor gene clusters encoding homologous Ole proteins but lack an *oleB* gene. Bacteria with this *oleADC* gene cluster create secreted β-lactone natural products, some of which are found to have antibiotic, anticancer, or antiobesity properties in medical testing (6). For example, salinosporamide A, a β-lactone natural product, is currently in phase three clinical trials for the treatment of glioblastoma (7). Lipstatin, produced by various *Streptomyces* spp., is hydrogenated industrially to make the FDA-approved antiobesity drug tetrahydrolipstatin (8, 9).

Both β-lactone and olefin pathways start with a nondecarboxylative Claisen condensation of acyl coenzyme A (acyl-CoA) precursors by OleA (Fig. 1), a member of the thiolase superfamily (10–12). Many thiolase enzymes catalyze carbon-carbon bond formation (13). They function in fatty acid, hydrocarbon, and natural product biosynthesis. As such, there is significant interest in identifying and reengineering these enzymes for biotechnological purposes (14). Our understanding thus far is derived almost exclusively from mechanistic and X-ray crystallographic studies with OleA from X. campestris (15–18) and a study on hydrocarbon biosynthesis in Micrococcus luteus (19). X. campestris OleA catalyzes the condensation of acyl-CoA substrates with C_10_-C_16_ acyl chains and produces long-chain hydrocarbons via deoxygenation reactions catalyzed by OleC and OleB proteins. Two distant homologs of OleA are LstA and LstB that together form a heterodimer in solution and catalyze the condensation of (3S,5Z,8Z)-3-hydroxytetradeca-5,8-dienoyl-CoA and octyl-CoA to produce the backbone of the β-lactone natural product, lipstatin (12).

The thiolase superfamily largely contains enzymes that join a short carbon chain to a growing chain, for making fatty acids or polyketide natural products (13). The singular characterized OleA differs from most thiolases in condensing two acyl chains ranging from C_8_ to C_16_ (11). However, the divergence in OleA and other thiolase sequences, as low as ∼17 to 30% amino acid identity, makes it currently impractical to predict function from sequence alone. To date, demonstrating a thiolase protein to be an OleA enzyme has required purifying the protein and carrying out a time-consuming assay. The reported assay for OleA activity is discontinuous and requires solvent extraction, gas chromatography, and calibration with authentic standard compounds that are not commercially available and require multistep syntheses (11, 13, 15). During the assay, the physiological β-keto acid product undergoes spontaneous decarboxylation, and the resultant ketone is quantified as a surrogate for the keto acid. This assay, as well as the poor solubility of these proteins, has precluded purification and characterization of OleA. Consequently, our current understanding of the biology and chemistry of OleA has largely been confined to the protein from X. campestris, although *in vivo* and bioinformatic analyses suggest that diverse bacterial strains produce OleA enzymes to make a wide range of different products (6, 10). The substrate specificity of OleA thus demonstrates the structures of the downstream products (Fig. 1). In this context, the identification and characterization of additional OleA enzymes provide the key to diverse natural products and membrane components generated by Ole proteins.

In the current work, we observed that the X. campestris and other OleA proteins will accept *p*-nitrophenyl alkanoates and catalyze a hydrolysis reaction to release the yellow product *p*-nitrophenol (*p-*NP). The tested thiolases FabH and Pks13 did not react, and the OleA reaction rates were surprisingly comparable to rates observed with lipases and other hydrolytic enzymes assayed with *p*-NP esters (20–23). This allowed the utility of *p*-NP ester reactivity to quantify OleA activity both *in vitro* and *in vivo*. The latter method, described here, served to rapidly screen 74 OleA homologs and identify and characterize new OleA proteins.

## RESULTS

### OleA enzymes are found in taxonomically diverse bacteria.

Despite the diversity of products made by Ole proteins (6, 10), previous efforts to purify five other OleA proteins were unsuccessful (11). This suggested that many OleA proteins are not amenable to purification and expression in heterologous hosts. In this context, genome sequences were analyzed here to identify divergent *oleA* genes that might produce stable and active OleA proteins when expressed heterologously in Escherichia coli.

**FIG 2:**
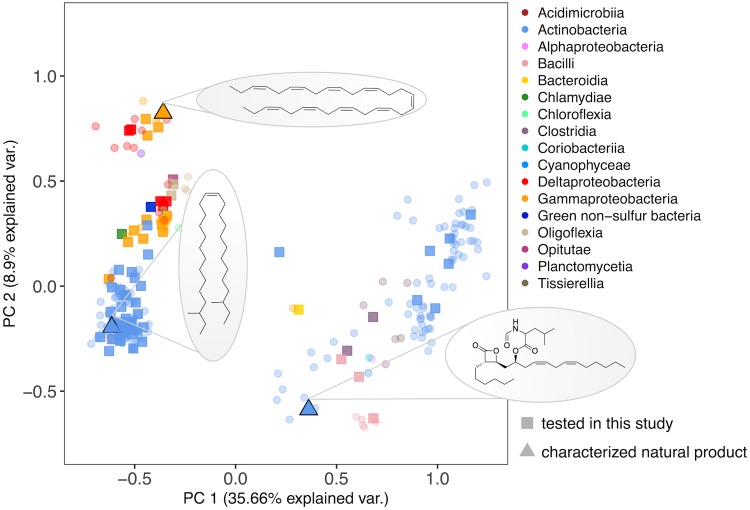
Sequence space of OleA proteins visualized using principal-coordinate analysis. The Whelan and Goldman distance matrix was used to calculate dissimilarity between full-length OleA amino acid sequences. Sequences are colored by taxonomic class. Circles represent 235 *oleA* homologs with flanking *oleCD* genes selected as cluster representatives from the thiolase superfamily (PF08545). Squares correspond to the 74 *oleA* genes that were synthesized and tested in this study. Triangles indicate OleA sequences for organisms with a structurally characterized Ole pathway product, a β-lactone or olefinic hydrocarbons, as shown in the ovals. var., variation.

In a broad screening effort, divergent OleA protein homologs were identified in 17 different taxonomic classes of bacteria, including *Chlamydiae*, *Clostridia*, *Opitutae*, and *Oligoflexia* (Fig. 2). Sequence cluster representatives belonging to 74 different organisms were selected as described in Materials and Methods to do the following: (i) define the sequence signature of true OleA proteins within the thiolase superfamily, (ii) screen for OleA proteins likely to express in active form in E. coli, and (iii) identify new OleA proteins that aid in determining the structural diversity of products made by OleA-initiated metabolic pathways. As is shown in Fig. 2, there are a limited number of characterized natural products that are associated with OleA proteins. Most of the proteins were annotated in GenBank as 3-oxo-acyl-acyl carrier protein (ACP) synthase III proteins. The amino acid sequence identities of the proteins screened here ranged from 24.3 to 87.9% to the X. campestris OleA amino acid sequence (see Table S1 in the supplemental material).

10.1128/mBio.00111-20.1 TABLE S1 Accession numbers, organism names, and percent amino acid sequence to X. campestris OleA of all OleA enzymes expressed and tested in the present assay. The percent amino acid sequence identity was calculated using BLAST. Download Table S1, PDF file, 0.1 MB. Copyright © 2020 Smith et al. 2020 Smith et al. This content is distributed under the terms of the Creative Commons Attribution 4.0 International license.

### Development of a colorimetric assay for OleA *in vitro*.

**FIG 3:**
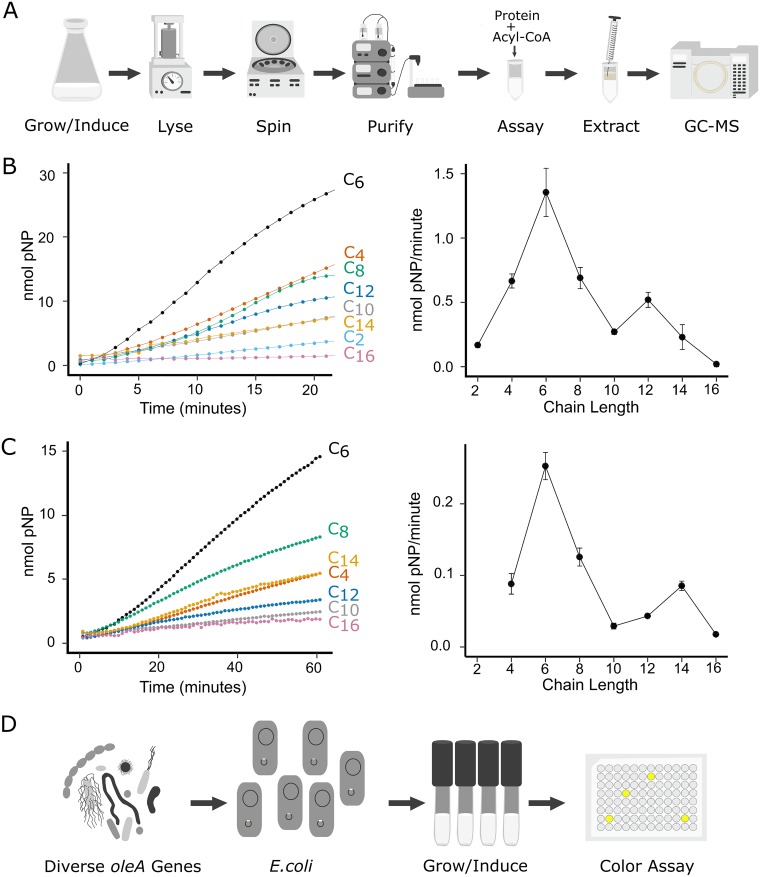
Assay schematic and data illustrating rapid assay compared to established methods to assay OleA activity. (A) The established OleA assay required purified proteins and expensive acyl-CoA substrates, reaction extractions, and gas chromatography-mass spectrometry (GC-MS) analysis of reaction products. (B) Assays conducted with purified OleA from Xanthomonas campestris using *p*-nitrophenyl acyl substrates of different chain lengths. (C) Whole-cell assays conducted with E. coli cells expressing X. campestris OleA and using *p*-nitrophenyl acyl substrates of different chain lengths. In panels B and C, rate curves are shown on the left and activity as a function of acyl chain length is shown on the right. (D) The rapid assay described in this paper screens diverse *oleA* genes, expressed recombinantly in E. coli cells using a colorimetric assay in microtiter well plates.

OleA proteins cannot be assayed in crude extracts with acyl-CoA substrates effectively due to interfering thioesterase activity, so studies thus far have been confined to *in vitro* experiments with purified enzymes (Fig. 3A). Over the course of *in vitro* coincubations with X. campestris OleA and porcine lipase, we observed unexpectedly high rates of hydrolysis of the lipase *p*-nitrophenyl ester substrate. Subsequently, it was determined that OleA alone catalyzed rapid color formation with *p*-nitrophenyl laurate. Indeed, when we compared rates of *p*-nitrophenyl ester hydrolysis of OleA compared to known hydrolases, OleA showed equivalent or greater rates (Table S2). OleA was shown to produce *p-*nitrophenol and a fatty acid via UV-visible (UV-Vis) spectroscopy and gas chromatography (see Fig. S1 in the supplemental material).

10.1128/mBio.00111-20.2 TABLE S2 Comparison of *in vitro* rates of *p*-nitrophenyl ester hydrolysis by OleA and other hydrolytic enzymes. Download Table S2, PDF file, 0.1 MB. Copyright © 2020 Smith et al. 2020 Smith et al. This content is distributed under the terms of the Creative Commons Attribution 4.0 International license.

10.1128/mBio.00111-20.4 FIG S1 Demonstration of hydrolysis of *p*-nitrophenyl laurate. (A) Release of *p*-nitrophenol demonstrated by UV/vis spectroscopy. OleA enzyme product (black curve), standard *p*-nitrophenol (gold curve), OleA enzyme product after the addition of HCl (blue curve), and *p*-nitrophenol standard after the addition of HCl (green curve) are shown. (B) Gas chromatograph of OleA reaction mixture or standard lauric acid after methylation by diazomethane. Download FIG S1, PDF file, 0.2 MB. Copyright © 2020 Smith et al. 2020 Smith et al. This content is distributed under the terms of the Creative Commons Attribution 4.0 International license.

Since *p*-nitrophenyl laurate has low water solubility and X. campestris OleA is promiscuous with respect to acyl-CoA chain length (C_10_ to C_16_), other *p*-nitrophenyl alkyl ester chain lengths were investigated. The reaction was observed to proceed similarly in microtiter wells or individual cuvettes, so microtiter plates were used in all subsequent experiments. Purified OleA was incubated individually with *p*-nitrophenyl esters containing all even-number carbon chain lengths ranging from C_2_ to C_16_, as shown in Fig. 3B. The reaction time course with C_2_ and C_4_ acyl chain lengths was linear from the first time points taken at less than 1 min. Longer chain length esters showed an initial lag phase before displaying a linear increase for 10 min or more. This has been observed previously in lipase assays and attributed to poor water solubility and longer dispersal times for the longer chain length esters (24). Maximum activity was observed with C_6_, followed by a linear decrease in activity with increasing chain length with the exception of a second smaller spike in activity at C_12_ (Fig. 3B). Given the poor solubility of the substrates, steady-state kinetic parameters could not be reliably measured, as previously discussed by Guthrie for *p*-nitrophenyl ester reactions with lipases (24). However, the observed rate with *p*-nitrophenyl hexanoate of 350 nmol per min per mg of protein was substantial, suggesting a potential for whole-cell screening of E. coli cells heterologously expressing different OleA proteins.

### Rapid *in vivo* screen for OleA.

On the basis of published reports (24–26), we expected that the long-chain *p*-nitrophenyl acyl esters would generally not enter E. coli cells without a permeabilizing agent. Following the general protocol for a high-throughput assay for oxygenases that used *p*-nitrophenyl ethers, polymyxin B sulfate was used as an E. coli cell permeabilizer (24, 25, 27). With this additive, rates for hydrolysis of C_4_ to C_16_ chain length *p*-nitrophenyl esters were significantly higher than the background, allowing a determination of OleA activity (Fig. 3C). Reaction rates with permeabilized cells per unit OleA protein estimated for the recombinant cells closely resembled the rates observed previously with purified enzyme. Moreover, the relative rates of different chain lengths followed a nearly identical pattern for purified enzyme (Fig. 3B) and permeabilized E. coli cells expressing OleA (Fig. 3C).

While the assay above is amenable to rapid screening, albeit with a 2-h preincubation to permeabilize cells, it would be faster and much more convenient if substrate could be added directly to cell suspensions without pretreatment. To test this, we used the highest-turnover substrate with moderate water solubility, *p*-nitrophenyl hexanoate. By direct addition of *p*-nitrophenyl hexanoate to cell suspensions in microtiter wells, yellow product formation over time could be monitored readily and at approximately 67% of the rate determined with permeabilized cells. In light of these results, the extensive purification and assay procedure (Fig. 3A) was replaced with a direct substrate drop-in assay with 96 reactions conducted simultaneously in microtiter plates (Fig. 3D). This assay was instrumental in identifying new, expressible, and active OleA enzymes.

### Screening of diverse bacterial thiolase proteins for identifying and obtaining new OleA proteins.

**FIG 4:**
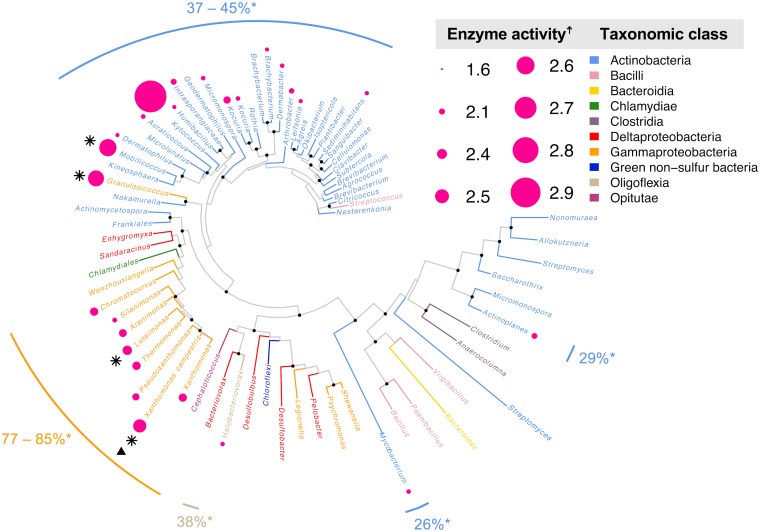
Phylogenetic tree of 73 taxonomically diverse OleA proteins assayed in this study using *p*-nitrophenyl hexanoate. †, Enzyme activity is shown as the log_10_ of nanomoles of pNP produced over the course of 1 h by an E. coli BL21 culture with an OD of 1.0 that is heterologously expressing OleA. Dark pink circles are scaled to relative enzyme activity levels measured in this study. Approximate maximum-likelihood phylogenetic analysis revealed three taxonomic classes of OleA homologs active with pNP esters: *Gammaproteobacteria* (orange), *Actinobacteria* (blue), and *Oligoflexus* (tan). Four of these proteins were purified to homogeneity and assayed with various chain length acyl-CoA substrates (black stars, see Fig. 5). Percentages marked with asterisks correspond to amino acid (aa) identity relative to X. campestris OleA (black triangle). Some actinobacterial sequences with as low as 26% aa identity to X. campestris are active with pNP esters. Branches are shown in color by the taxonomic class of the source organism. Black nodes correspond to branch points with probabilities of > 0.75.

Seventy-four recombinant E. coli strains containing genes for putative OleA proteins were screened *in vivo* using direct addition of *p*-nitrophenyl hexanoate (Fig. 4). Hydrolysis rates for recombinant cells were normalized against E. coli containing an empty vector, and rates for positively increasing slopes were recorded (Fig. S2). In order to remove potential false-positive results, only those with a rate above the mean activity were recorded as active (Fig. S3). The final compiled rates for all proteins screened across three biological replicates (Table S3) were mapped onto a phylogenetic tree of OleA sequences (Fig. 4). Phylogenetic analysis revealed two distinct clades of actinobacterial OleA sequences with one clade displaying significant activity and the other displaying only weak activity in one homolog (Fig. 4). In total, excluding our positive-control OleA from X. campestris, 25 OleA proteins were found to be active, representing ∼35% of the proteins screened for activity. This proportion is an increase to our previous experience with OleA proteins in which one in five could be produced as active enzymes in *E coli* (11).

10.1128/mBio.00111-20.3 TABLE S3 Average enzyme activity (log_10_ of nanomoles pNP produced over the course of 1 h by an E. coli BL21 culture with an OD of 1.0) for 73 OleA enzymes. Download Table S3, PDF file, 0.2 MB. Copyright © 2020 Smith et al. 2020 Smith et al. This content is distributed under the terms of the Creative Commons Attribution 4.0 International license.

10.1128/mBio.00111-20.5 FIG S2 Heatmap of relative enzyme activity (log_10_ of nanomoles of pNP produced over the course of one hour by a E. coli BL21 culture with an OD of 1.0) of 73 OleA enzymes across three different replicates. Enzyme activity ranges from high activity (yellow) to no activity (dark purple). Results reveal general reproducibility of this whole-cell method for enzymes with high activity and lower reproducibility for enzymes with weak activity. Download FIG S2, PDF file, 0.1 MB. Copyright © 2020 Smith et al. 2020 Smith et al. This content is distributed under the terms of the Creative Commons Attribution 4.0 International license.

10.1128/mBio.00111-20.6 FIG S3 Graph of active OleA enzymes in whole-cell *p*-NP assays across all three replicates, along with a reference line for calculated slope. Active organisms for each replicate are listed besides the table as well as the rate of enzyme activity in nanomoles of *p*-NP/OD of 1.0/hour. Download FIG S3, PDF file, 0.2 MB. Copyright © 2020 Smith et al. 2020 Smith et al. This content is distributed under the terms of the Creative Commons Attribution 4.0 International license.

There were clearly two major taxonomic clades that yielded active OleA proteins at a higher frequency than the cumulative 35% success rate. Indeed, the gammaproteobacterial proteins that were most similar (77 to 88% identity [ID]) to the X. campestris OleA were almost uniformly reactive with *p*-nitrophenyl hexanoate, giving an 88% success rate. However, in that group, none showed a higher activity than the characterized X. campestris OleA. Interestingly, a cluster of actinobacterial OleA proteins, and a related gammaproteobacterial sequence from *Granulosicoccus*, showed significantly higher activity than any of the proteins clustering with the X. campestris OleA (Fig. 4). The highest activity overall was observed with an OleA homolog from Kytococcus sedentarius, a bacterium isolated from a marine environment in 1944, but also commonly found on human skin (28). The second highest activity was observed with an OleA from Mobilicoccus massiliensis, a bacterium isolated from a human stool sample (29). The *M. massiliensis* OleA amino acid sequence is only 37% identical to that from X. campestris, and the *Kytococcus* OleA amino acid sequence is likewise only 45% identical to the X. campestris OleA. *Kytococcus* and *Mobilicoccus* are both members of the order *Micrococcales*. The taxonomic outlier represented in this cluster of OleA sequence space is Granulosicoccus antarcticus, which is a marine gammaproteobacterium (30). The sequence of the *Granulosicoccus* OleA is fairly divergent, showing only 40 to 51% amino acid sequence identity to the other highly active proteins from *Xanthomonas*, *Mobilicoccus*, and *Kytococcus*. Several other actinobacterial OleA proteins showed activity, including proteins from Actinoplanes atraurantiacus, and Mycobacterium obuense. A. atraurantiacus is a bacterium isolated from forest soil (31), and from a completely different family of *Actinobacteria*, the *Micromonosporaceae*, that are known for their prolific production of secondary metabolites (32). Mycobacterium obuense is a nonpathogenic member of the *Mycobacterium* genus that is studied for potential use in bioremediation (33). Another active OleA is from Halobacteriovorax marinus, belonging to the *Oligoflexia* class, found in estuaries and known to prey on Gram-negative bacteria (34). The *Actinoplanes* and *Mycobacterium* proteins are the most divergent active proteins from the X. campestris OleA, showing only 29% and 26% amino acid sequence identity, respectively. In light of these large sequence and taxonomic differences, we chose selected proteins to confirm that they showed Claisen condensation activity with long-chain acyl-CoA substrates, characteristic of OleA proteins, and to further explore gene cluster differences, both of which provide insights into the biological function of these OleA proteins.

### Purification of new OleA proteins and investigating Claisen reactivity.

Reaction of the *p*-nitrophenyl acyl substrates with OleA proteins leads to two products, a fatty acid and *p*-nitrophenol. We have not detected evidence of Claisen condensation between acyl chains of *p*-nitrophenyl esters as observed with native acyl-CoA substrates. However, purified X. campestris OleA mutants in which the active site cysteine is mutated to a serine or alanine do not have *p*-nitrophenyl ester hydrolysis activity (Fig. S3). This indicated that the acyl chain is undergoing a transesterification from the activated *p*-nitrophenyl ester to the active site cysteine, comparable to the transesterification from acyl coenzyme A to the cysteine in the physiological reaction. Analysis of the reaction of OleA with *p*-nitrophenyl esters of various chain lengths using gas chromatography (GC)-mass spectrometry (MS) did not produce any detectable β-keto acid product. Presumably, hydrolysis of the enzyme intermediate outcompetes the binding and subsequent Claisen condensation with a second acyl substrate. In this context, we sought to determine whether the newly identified proteins would indeed catalyze a Claisen condensation reaction with acyl-CoA substrates.

**FIG 5:**
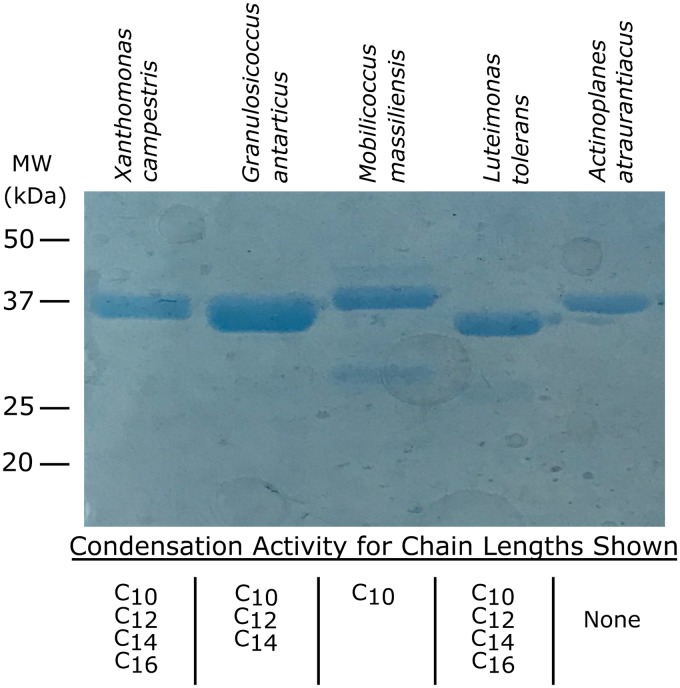
Purification, SDS-PAGE analysis, and assay of OleA proteins for Claisen condensation activity with long-chain acyl-CoA substrates. Each protein was assayed for Claisen condensation reactivity with acyl-CoA substrates ranging from C_8_ to C_16_ as described in Materials and Methods. The chains lengths that reacted are indicated at the bottom. MW, molecular weight.

To test this, several proteins were purified and assayed via the standard assay procedure (Fig. 5). We selected recombinant E. coli clones expressing highly active OleA proteins from the genera *Granulosicoccus*, *Mobilicoccus*, and *Luteimonas*. We also chose the *Actinoplanes* OleA protein, as it was one of the most divergent with respect to sequence and the taxonomy of the native organism. The first three proteins were stable to purification via nickel affinity column chromatography. However, the *Actinoplanes* enzyme was produced in low yield and readily precipitated upon concentration. The proteins expressed well in and showed expected bands of ∼37-kDa subunit molecular weights when analyzed by sodium dodecyl sulfate-polyacrylamide gel electrophoresis (SDS-PAGE) (Fig. 5).

Enzyme assays were run via the standard assay involving incubation, extraction, and GC-MS analysis to detect and confirm the expected Claisen condensation product (Fig. 5). X. campestris OleA served as a positive control, and negative controls consisted of blanks without enzyme. The positive control condensed acyl-CoA with chain lengths of C_10_, C_12_, C_14_, and C_16_, similar to previous experiments (11). The *Granulosicoccus* OleA condensed C_10_, C_12_, and C_14_ chains, the *Mobilicoccus* Ole condensed C_10_ chains, and the *Luteimonas* OleA condensed C_10_, C_12_, C_14_, and C_16_ chains, similar to the *Xanthomonas* enzyme. Limited *Actinoplanes* enzyme was available due to precipitation during purification, and the recoverable protein did not react with any of the acyl-CoA substrates tested.

### Gene context and biological function of OleA proteins.

**FIG 6:**
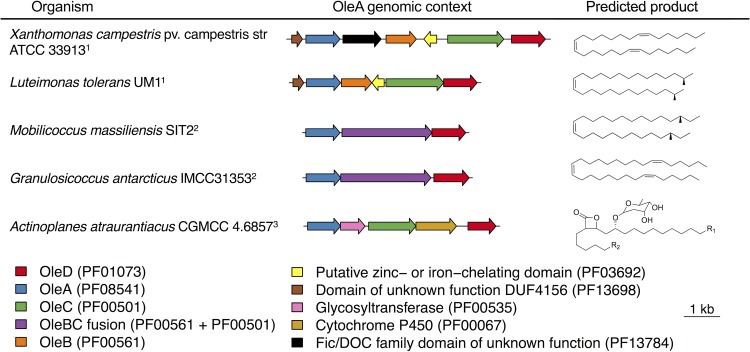
Genomic context for genes encoding OleA proteins that were expressed and purified in this study. Coloring corresponds to PFAM domain(s) present in each gene cluster. Analysis revealed three types of genome contexts: ^1^*oleABCD* clusters with 0 to 3 intermediate genes, ^2^*oleBC* fusion genes in *oleABCD* clusters, and ^3^*oleACD* clusters lacking *oleB* homologs likely making β-lactone natural products (e.g., lipstatin analogs). Predicted products are inferred based on biosynthetic gene cluster synteny with known natural products, the predominant fatty acid composition of source organisms, and functional knowledge of the protein domains flanking OleA.

The biological products from OleA-initiated metabolic pathways may be inferred from their genomic context and the fatty acyl chain pool of the native, producing organism (Fig. 6). Xanthomonas campestris condenses a number of saturated and unsaturated fatty acids to make long-chain olefinic hydrocarbons, shown previously by GC-MS analysis of membrane extracts (10). The X. campestris genome region contains two additional genes encoding Pfam domains PF13784 and PF03692, respectively, in addition to the *oleABCD* genes encoding proteins with known function. The related gammaproteobacterial protein from *Luteimonas* has a similar *ole* gene region but is lacking the PF13784 domain. The significance of these domains is currently unknown. On the basis of previous work showing that major fatty acid types are condensed to form olefins (35), we predict that branched-chain hydrocarbons are formed by *Luteimonas* (Fig. 6). *Mobilicoccus* and *Granulosicoccus* have similar gene regions, but somewhat different membrane hydrocarbons are predicted based on differences in fatty acid content. A major feature of both of those gene regions is the presence of an *oleBC* gene fusion that tethers the OleB and OleC enzyme activities together into one polypeptide (4). This has been observed previously to occur in certain *Actinobacteria* typically in the order *Micrococcales*. The presence of the *oleBC* gene fusion in *Granulosicoccus* is the first observation of this outside the *Actinobacteria*, and the similar gene architecture may in fact be reflective of a horizontal gene transfer event.

The A. atraurantiacus gene region is the most different, and we believe that it reflects a different biological function for the *ole* genes. The entire *A. atraurantiacus* genome lacks a candidate *oleB* gene. OleB makes a β-lactone decarboxylase enzyme (5) that acts in the membrane biosynthesis pathway to transform β-lactones into an olefinic hydrocarbon (Fig. 1). The absence of this gene infers that the final product is a β-lactone. Indeed, other *Actinobacteria*, such as Streptomyces toxytricini and Nocardia brasiliensis that have *oleACD* biosynthetic gene clusters but lack an *oleB* gene produce β-lactone natural products (8, 36). The annotated gene region from the *A. atraurantiacus* genome indicates the following protein domains: OleA, a glycosyltransferase, OleC, a cytochrome P450, and an OleD. That cluster of protein domains suggests a Claisen condensation of fatty acyl groups (OleA), reduction of the condensed product (OleD), hydroxylation of one of the acyl chains (cytochrome P450), glycosylation of the alcohol (transferase), and ring closure to make the β-lactone (OleC). The exact positions of the substituents cannot be determined, but the structure shown in Fig. 6 is representative of the type of product that could be made by *A. atraurantiacus.*

## DISCUSSION

An impediment to more broadly studying diverse OleA proteins has been their poor expression/activity in E. coli and the slow published assay that required fixed time point sampling, extraction, and chromatography. This was overcome in the present study by the discovery and standardization of a rapid, sensitive, color-based assay. Many proteins have been shown to catalyze hydrolysis of *p*-nitrophenyl esters (20–24, 37), but to our knowledge, the Ole proteins are the first members of the thiolase superfamily to perform this reaction. For a control, we tested here the thiolase enzymes Pks13 from Mycobacterium tuberculosis and FabH from E. coli with *p*-nitrophenyl hexanoate and found no discernible reactivity. This helps explain the low background activity of E. coli enabling the development of a whole-cell assay. Most reported enzymes reacting with *p*-nitrophenyl esters are serine hydrolases such as lipases and proteases. Porcine intestinal lipase was tested here and showed only 13% of the specific activity observed with OleA when assayed with *p*-nitrophenyl hexanoate (see Table S2 in the supplemental material). Many lipases and other reactive serine enzymes are assayed with *p*-nitrophenyl acetate, which is much more soluble than its longer-chain counterparts. However, this substrate was not suitable for the *in vivo* assay developed here, as *p*-nitrophenyl acetate gave a very high background rate of hydrolysis in wild-type E. coli cells. In order to mitigate against false-positive results, we included only proteins showing a significant, reproducible level of activity, as discussed in Materials and Methods. The strict criteria used here eliminated consideration of a significant number of OleA proteins with low activity. Indeed, based on the heatmap shown in Fig. S2 in the supplemental material, more than two thirds of the OleA enzymes tested showed some measurable level of activity above background. Given that OleA proteins in divergent bacteria produce different products (10) (Fig. 6), it is not surprising that some enzymes would not optimally bind and react with *p*-nitrophenyl hexanoate. Using positive results from the screen that fell within the cutoff, we had a 75% success rate of purification of our four chosen enzymes, which is much higher than the 20% success rate for five candidate enzymes examined in previous research (11).

The high rate of OleA in hydrolyzing *p*-nitrophenyl hexanoate versus other E. coli proteins allowed for a rapid assay to screen a wide range of putative OleA proteins that could be identified by bioinformatics. The only well-characterized OleA thus far has been the protein from X. campestris, a homodimer in which a glutamate from one subunit acts as a general base in the active site of the second subunit. This mechanism is unique to OleA homologs compared to other characterized proteins in the thiolase superfamily. A multiple-sequence alignment of the OleA proteins showed that all of the proteins except two showed a glutamate at position 117 followed by a proline (Fig. S5). This glutamate was found to be essential to the Claisen condensation in X. campestris OleA. Bioinformatic analysis, both published (10) and conducted here, indicate that there are more than one thousand such OleA-type proteins in GenBank likely involved in natural product or membrane biosynthesis. The number and diversity of operons harboring *oleA* genes across different phyla is expansive, suggesting that the substrate specificity of these proteins and the final pathway products extend well beyond what has been characterized thus far. Indeed, even with our strict cutoff, we saw activity with OleA proteins from A. atraurantiacus and M. obuense. These proteins are 29% and 26% identical to OleA from Xanthomonas campestris. For a comparison, FabH from E. coli has a 27% sequence identity to X. campestris OleA yet showed no activity with *p*-nitrophenyl hexanoate. Additionally, although we were able to purify *A. actinoplanes* OleA, it quickly precipitated out of solution and was difficult to work with. These two pieces of data highlight two major points of the assay. One is that we can use the first transesterification step to test for activity for OleA enzymes from even a wide range of diversity. Second, this general method may be used to study OleA activity with enzymes that may not be amenable to purification. This opens up a much greater diversity of OleA enzymes to study.

OleA enzymes with different substrate selectivity can be combined with broad-specificity OleD and OleC enzymes to generate diverse β-lactones, which are of interest for their medicinal properties. In this context, it would be very beneficial if *p*-nitrophenyl esters could substitute for acyl-CoA compounds in biocatalytic cascades for making β-lactones. *p*-Nitrophenyl esters are commercially available, relatively inexpensive, and easy to synthesize compared to their CoA counterparts. However, we have not observed Claisen condensation to occur with *p*-nitrophenyl alkanoates and the OleA enzymes. The reason is not immediately clear, since there is evidence that the reaction initiates similarly to the acyl-CoA reaction with transfer of the acyl group to the active site cysteine. When C-143 is mutated to an alanine or when OleA is incubated with cerulenin, an inhibitor shown to interact with the active site cysteine (15), *p*-NP hydrolysis does not occur above background levels (Fig. S4). This is similar to the change in rate of CoA hydrolysis from acyl-CoA substrates (16). Studies investigating the potential OleA-catalyzed Claisen condensation of *p*-nitrophenyl alkanoates are under way.

10.1128/mBio.00111-20.7 FIG S4 4 μg of wild-type (WT) OleA (green) and OleA C143A (magenta) and WT with 25 μM cerulenin (blue) were assayed with 200 μM *p*-nitrophenyl dodecanoate in 50 mM Tris HCl (pH 8) at 37°C. Cerulenin was added prior to the assay, and the plate was allowed to incubate for 10 min at 37°C. Download FIG S4, PDF file, 0.1 MB. Copyright © 2020 Smith et al. 2020 Smith et al. This content is distributed under the terms of the Creative Commons Attribution 4.0 International license.

10.1128/mBio.00111-20.8 FIG S5 Structure-based multiple-sequence alignment of OleA proteins screened in this study. The numbering and structural elements depicted are based on the X-ray structure of the X. campestris OleA. Glutamate 117 is highly conserved. Proline 118 and cysteine 143 are completely conserved. Download FIG S5, PDF file, 0.7 MB. Copyright © 2020 Smith et al. 2020 Smith et al. This content is distributed under the terms of the Creative Commons Attribution 4.0 International license.

Overall, this new assay allows for rapid analysis of libraries of diverse OleA homologs. This has implications not only for finding other novel β-lactones in nature but also for finding novel bioproducts such as surfactants for which pathways are initiated by OleA. Additionally, this provides a solid basis for understanding OleA proteins for enzyme engineering to broaden substrate specificity. While this study utilized *p*-nitrophenyl hexanoate, one could imagine screening a similar library using *p*-NP esters that are very different from the natural alkyl chain substrates, leading to the biosynthesis of novel β-lactones.

## MATERIALS AND METHODS

### Chemicals and reagents.

The following *p*-nitrophenyl acyl esters were obtained from Sigma-Aldrich (St. Louis, MO): acetate, butyrate, octanoate, decanoate, myristate, and palmitate. *p-*Nitrophenyl hexanoate was obtained from Tokyo Chemical Industry. Coenzyme A, Tris-HCl, *p*-nitrophenol, lysogeny broth mix, granulated agar, and methyl tertiary-butyl ether were also obtained from Sigma-Aldrich. Polymyxin B sulfate was obtained from Alfa Aesar. Isopropyl-β-d-1-thiogalactopyranoside (IPTG) and kanamycin were obtained from GoldBio.

### Computational methods and phylogenetics.

Seed sequences were selected from 16 known and highly likely OleA enzymes from genes in organisms producing long-chain olefin products. The sequences were structurally aligned using T-Coffee Expresso (38) and used to build a profile hidden Markov model (pHMM) specific for OleA enzymes using HMMER3 (39). The pHMM was searched against a custom database of 47,093 nonredundant RefSeq protein sequences containing at least one 3-oxoacyl-acyl carrier protein synthase III domain (PF08541). The pHMM hits were trimmed to a stringent E-value cutoff of 1e^−42^ to yield 920 unique OleA-like protein sequences. The flanking genes within a six-gene window on either side of each *oleA* homolog were pulled using RODEO (40). There were 251 *oleA* homologs with at least two flanking pathway genes (*oleB*, *oleC*, or *oleD*) within the same gene neighborhood as determined by pHMM. These 251 “high-confidence” *oleA* sequences with flanking hydrocarbon or β-lactone biosynthetic genes were further filtered to include only sequences with a length of 500 amino acids or less. The remaining 234 sequences were then clustered using CD-Hit at 50% sequence identity cutoff with a word size of 2 to obtain 41 cluster representatives. Of these, 27 *oleA* genes were selected on the basis of taxonomic diversity. The remaining 46 *oleA* genes were manually selected. To conduct phylogenetic analysis, amino acid sequences for 234 candidate OleA proteins were aligned using DECIPHER (41). The alignment was trimmed, and FastTree was used with default parameters to infer the approximate maximum-likelihood phylogeny using the Jones-Taylor Thornton model with CAT approximation (42).

### Bacterial strains and growth conditions.

E. coli T7 strains (catalog no. C2566I; New England BioLabs [NEB]) were used that contained a pET-28b+ vector. One strain was a vector-only control, and another contained *oleA* from Xanthomonas campestris (NP_635607.1). Homologs of OleA found in other strains were synthesized, placed into a pET-28b+ vector, and transformed into T7 Express Competent E. coli by the Joint Genome Institute. Cells were grown at 37°C in lysogeny broth medium until an optical density (OD) of 0.3. They were then induced with 1 mM IPTG and incubated at 16°C overnight. Experiments using these cells follow the protocols below.

### *In vitro* plate assay for OleA.

Tris-HCl (50 mM) (pH 8.0) was added to a 96-well flat-bottom suspension culture plate (catalog no. 25-104; Genesee Scientific) containing 4 μg of OleA, 5% ethanol, and 200 μM *p*-NP alkanoate in a total volume of 200 μl. Absorbance was read at 410 nm every minute for 30 min at 37°C. A standard curve was developed in a buffer containing 50 mM Tris-HCl at pH 8.0. To precisely determine the extinction coefficient for *p*-nitrophenol under the conditions used here, commercially available *p*-nitrophenol was added in different concentrations to 50 mM Tris-HCl (pH 8.0) and 5% ethanol. Absorbance was read at 410 nm in cuvettes with a 1-cm path length in a SpectraMax Plus 384 microplate reader (Molecular Devices). The path length for the 200-μl reaction mixture in 96-well plates was determined by adding known concentrations of the same commercially available *p*-nitrophenol to the 50 mM Tris-HCl (pH 8.0) buffer with 5% ethanol and using the calculated extinction coefficient and absorbance read at 410 nm to determine the path length to be 0.58 cm. The extinction coefficient under these conditions was determined to be 15,546 M^−1 ^cm^−1^, and this value was used for subsequent calculations. All reactions were run in parallel on the same plates as three to five replicates for data determination. Controls of each *p-*nitrophenyl alkanoate chain length containing no protein were used to normalize against any nonenzymatic hydrolysis in buffer.

### *In vivo* plate assay for OleA.

E. coli BL21(DE3) cells were transformed with either empty pET-28b+ vector or the vector containing an *oleA* gene. Cells were induced with 100 μM isopropyl-β-d-thiogalactopyranoside when the absorbance reached 0.3 (600 nm). Induced cells were grown overnight at 16°C followed by resuspension in 50 mM Tris-HCl (pH 8.0) buffer at 0.1 OD. Resuspended cells were then diluted 16-fold and added to a 96-well microtiter plate containing 5% ethanol and 200 μM *p-*nitrophenyl alkanoate for a total volume of 200 μl. Absorbance was read every minute for 60 min at 410 nm at 37°C. Absorbance was normalized to induced cells containing empty pET-28b+ vector to account for any hydrolysis of the respective *p-*nitrophenyl alkanoate in buffer plus hydrolysis by any other E. coli enzymes other than OleA.

The use of cell lysis reagents and solubilizers of long-chain *p*-nitrophenyl esters was also tested to determine the effects on the observed rates. In those experiments, 63 μM polymyxin B sulfate and 10 mM methyl-β-cyclodextrin were added to cell suspension in the wells and allowed to incubate at room temperature for 2 h before the addition of *p*-NP.

### Data analysis.

Absorbance values were normalized by subtracting the absorbance values for cells with an empty pET-28b+ vector control. Absorbance was converted to nanomoles of *p*-NP produced by an E. coli BL21 culture with an OD of 1.0 using the Beer-Lambert law (ε_410_ = 15,546 M^−1^ cm^−1^). Slopes were calculated using a rolling linear regression window method by calculating slopes for all overlapping 15-min intervals over the course of the first 45 min of each reaction. The greatest slope for each enzyme with *R*^2^ ≥ 0.9 was selected as maximum enzyme activity for a given OleA (nanomoles of *p*-NP/OD of 1.0/hour). Enzyme activity values across triplicate measurements were averaged. Activity values displayed a right skewed distribution; therefore, a log_10_ transformation was applied for downstream analysis, resulting in an approximately normal distribution. Outliers more than 1.5 interquartile ranges (IQRs) below the first quartile or above the third quartile were removed to calculate mean enzyme activity. To set a stringent threshold for activity and filter out false-positive results, we labeled enzymes that were greater than or equal to the mean enzyme activity of 1.66 log_10_ nmol *p*-NP/OD of 1.0/hour) as “active,” while those below the mean level of activity were considered “inactive.”

### Protein purification and characterization.

OleA proteins were purified, and assays with acyl-CoA compounds were performed as previously described for *X*, *campestris* OleA (11).

10.1128/mBio.00111-20.9 FIG S6 (A) GC-flame ionization detector (FID)/MS split chromatogram of an extract from reaction of OleA proteins from different sources with decanoyl-CoA showing 10-nonadecanone, the stable decarboxylated product of the Claisen condensation that can be detected by GC. *A. atraurantiacus* (black), *M. massiliensis* (orange), *L. tolerans* (blue), and *G. antarticus* (green) are shown. The inset shows the MS spectrum of the peak (from the representative Luteimonas tolerans OleA). All mass spectra from reactions showing a peak gave the characteristic fragments for 10-noadecanone. (B) GC-FID/MS split chromatogram of an extract from reaction of OleA proteins from different sources with lauryl-CoA showing 12-tricosanone, the stable decarboxylated product of the Claisen condensation that can be detected by GC. *A. atraurantiacus* (black), *M. massiliensis* (orange), *L. tolerans* (blue), and *G. antarticus* (green) are shown. The inset shows the MS spectrum of the eluted standard peak. All mass spectra from reactions showing a peak gave the characteristic fragments for 12-tricosanone. Download FIG S6, PDF file, 0.1 MB. Copyright © 2020 Smith et al. 2020 Smith et al. This content is distributed under the terms of the Creative Commons Attribution 4.0 International license.

10.1128/mBio.00111-20.10 FIG S7 (A) GC-FID/MS split chromatogram of an extract from reaction of OleA proteins from different sources with myristoyl-CoA showing 14-heptacosanone, the stable decarboxylated product of the Claisen condensation that can be detected by GC. *M. massiliensis* (orange), *L. tolerans* (blue), *G. antarticus* (green), and the chemical standard (gray) are shown. *A. atraurantiacus* was not included due to inactivity and insolubility. The inset shows the MS spectrum of the eluted standard peak. All mass spectra from reactions showing a peak gave the characteristic fragments for 14-heptacosanone. (B) GC-FID/MS split chromatogram of an extract from reaction of OleA proteins from different sources with palmitoyl-CoA showing 16-hentriacontanone, the stable decarboxylated product of the Claisen condensation that can be detected by GC. *M. massiliensis* (orange), *L. tolerans* (blue), *G. antarticus* (green), and the chemical standard (gray) are shown. *A. atraurantiacus* was not included due to inactivity and insolubility. The inset shows the MS spectrum of the eluted standard peak. All mass spectra from reactions showing a peak gave the characteristic fragments for 16-hentriacontanone. Download FIG S7, PDF file, 0.1 MB. Copyright © 2020 Smith et al. 2020 Smith et al. This content is distributed under the terms of the Creative Commons Attribution 4.0 International license.
