# Supplementary material for: In Vivo Assay Reveals Microbial OleA Thiolases Initiating Hydrocarbon and β-Lactone Biosynthesis
Source: mBio. 2020 Mar 10;11(2):e00111-20. doi: 10.1128/mBio.00111-20 (PMC7064751; doi:10.1128/mBio.00111-20)
Supplement: TABLE S1 [file mBio.00111-20-st001.pdf]

Accession Number	Organism Name	%ID to X. campestris OleA
WP_003480168	*Xanthomonas_translucens*	87.9
WP_130552392	*Pseudoxanthomonas_sp.*	87.3
WP_022969495	*Arenimonas_oryziterrae*	80.8
WP_114959563	*Thermomonas_haemolytica*	79.9
WP_028771085	*Silanimonas_lenta*	77.8
WP_076585679	*Luteimonas_tolerans*	77.2
WP_117315975	*Chromatocurvus_halotolerans*	59.8
WP_116651321	*Wenzhouxiangella_sediminis*	58.6
WP_053234550	*Sandaracinus_amylolyticus*	53.0
WP_106091803	*Enhygromyxa_salina*	52.1
WP_119951226	*Frankiales_bacterium*	49.1
WP_088206054	*Chlamydiales_bacterium*	46.2
WP_013837355	*Isoptericola_variabilis*	45.5
WP_101620581	*Brevibacterium_antiquum*	45.4
WP_079726385	*Okibacterium_fritillariae*	45.2
WP_012802190	*Kytococcus_sedentarius*	45.2
WP_026204600	*Actinomycetospora_chiangmaiensis*	44.8
WP_100422947	*Sediminihabitans_luteus*	44.4
WP_026862459	*Intrasporangiaceae_bacterium*	44.3
WP_015490789	*Clavibacter_michiganensis*	43.9
WP_070193933	*Humibacillus_sp.*	43.7
WP_012868615	*Sanguibacter_keddieii*	43.6
WP_044441590	*Agreia_bicolorata*	43.3
WP_086990725	*Agrococcus_casei*	43.0
WP_090596165	*Auraticoccus_monumenti*	42.9
WP_013865923	*Microlunatus_phosphovorus*	42.9
WP_012948569	*Geodermatophilus_obscurus*	42.9
WP_091632192	*Micromonospora_peucetia*	42.6
WP_034630949	*Cellulomonas_cellasea*	42.4
WP_056164922	*Leifsonia_sp.*	42.4
WP_088919872	*Granulosicoccus_antarcticus*	42.3
WP_003805730	*Arthrobacter_globiformis*	42.2
WP_086472704	*Plantibacter_sp.*	42.0
WP_033420292	*Nesterenkonia_alba*	41.1
WP_005885358	*Brevibacterium_mcbrellneri*	40.9
WP_104244666	*Subtercola_sp.*	40.6

Accession Number	Organism Name	%ID to X. campestris OleA
WP_101686474	*Dermabacter_hominis*	40.5
WP_058859095	*Kocuria_flava*	39.6
WP_126985334	*Brachybacterium_paraconglomeratum*	39.6
WP_068469535	*Kocuria_varians*	39.4
WP_015749354	*Nakamurella_multipartita*	39.3
CVN04163	*Streptococcus_pneumoniae*	38.8
WP_115933252	*Citricoccus_muralis*	38.6
WP_096909276	*Halobacteriovorax_marinus*	38.2
WP_068712879	*Cephaloticoccus_capnophilus*	38.1
WP_096166078	*Brachybacterium_alimentarium*	38.0
WP_028327376	*Dermatophilus_congolensis*	37.8
WP_040156198	*Mobilicoccus_massiliensis*	37.4
OGN97459	*Chloroflexi_bacterium*	37.0
WP_005508112	*Rothia_mucilaginosa*	36.9
WP_006592558	*Kineosphaera_limosa*	36.8
WP_021267235	*Bacteriovorax_sp.*	36.6
WP_051554118	*Desulfobulbus_elongatus*	36.6
ABL00697	*Pelobacter_propionicus*	36.1
WP_028865527	*Psychromonas_aquimarina*	35.0
WP_061782733	*Shewanella_putrefaciens*	34.1
WP_058441655	*Legionella_brunensis*	32.6
WP_020589018	*Desulfobacter_curvatus*	32.5
WP_030433686	*Allokutzneria_albata*	29.3
WP_097326105	*Actinoplanes_atraurantiacus*	29.1
WP_053141838	*Streptomyces_ambofaciens*	29.0
WP_076469753	*Micromonospora_avicenniae*	28.3
WP_010983715	*Streptomyces_avermitilis*	27.1
WP_099839552	*Clostridium_combesii*	27.0
WP_015101236	*Saccharothrix_espanaensis*	26.5
WP_082133371	*Mycobacterium_obuense*	25.9
WP_092493676	*Virgibacillus_salinus*	25.4
WP_090922125	*Paenibacillus_polysaccharolyticus*	25.1
WP_063765283	*Nonomuraea_candida*	24.9
WP_084558798	*Anaerocolumna_xylanovorans*	24.8
WP_073399371	*Bacteroides_luti*	24.8
WP_095301808	*Bacillus_sp.*	24.3

Table S1. Accession number, organism name, and percent amino acid sequence to *X. campestris* OleA of all OleAs expressed and tested in the present assay. The percent amino acid sequence identity was calculated using BLAST.
