# Supplementary material for: In Vivo Assay Reveals Microbial OleA Thiolases Initiating Hydrocarbon and β-Lactone Biosynthesis
Source: mBio. 2020 Mar 10;11(2):e00111-20. doi: 10.1128/mBio.00111-20 (PMC7064751; doi:10.1128/mBio.00111-20)
Supplement: TABLE S2 [file mBio.00111-20-st002.pdf]

Table S2. Comparison of *in vitro* rates of *p*-nitrophenyl ester hydrolysis by OleA and other hydrolytic enzymes

Enzyme	Source	(Pfam ID)	Protein family	Reaction	Active Site Nucleophile	Relative Rate (%)		
						C6	C10	C14
OleA (β-keto acid synthase)	*Xanthomonas campestris*	(PF08545)	3-Oxoacyl-ACP synthase III	Transferase (Synthase)	Cysteine	100	100	100
Intestinal Lipase	*Sus domesticus*	(PF00151)	Triglyceride Lipase	Ester Hydrolysis	Serine	13	89	8
Papain	*Ananas comosus*	(PF00112)	Peptidase C1	Amide Hydrolysis	Cysteine	<1	<1	<1
Chymotrypsin-α	*Sus domesticus*	(PF00089)	Trypsin	Amide Hydrolysis	Serine	100	8	4
Arylaceto-nitrilase	*Alcaligenes faecalis*	(PF00795)	CN Hydrolase	Nitrile Hydration	Cysteine	2	<1	7
