# Supplementary material for: In Vivo Assay Reveals Microbial OleA Thiolases Initiating Hydrocarbon and β-Lactone Biosynthesis
Source: mBio. 2020 Mar 10;11(2):e00111-20. doi: 10.1128/mBio.00111-20 (PMC7064751; doi:10.1128/mBio.00111-20)
Supplement: FIG S1 [file mBio.00111-20-sf001.pdf]

A

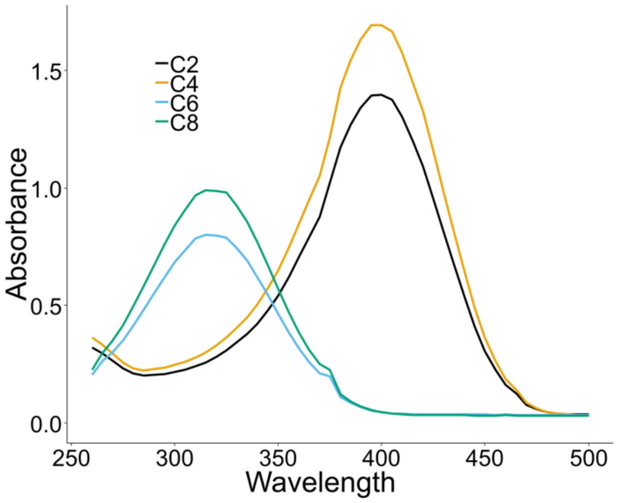

B

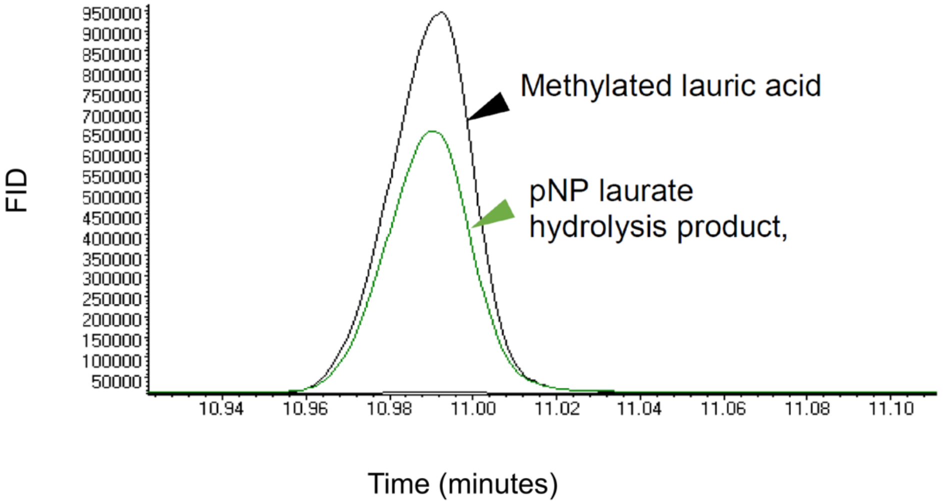

Figure S1. Demonstration of hydrolysis of *p*-nitrophenyl laurate. (A) Release of *p*-nitrophenol demonstrated by UV/vis spectroscopy; black curve of OleA enzyme product, gold curve is standard *p*-nitrophenol; blue curve is the OleA enzyme product after addition of HCl and green is the *p*-nitrophenol standard after HCl addition. (B) Gas chromatograph of OleA reaction mixture or standard lauric acid after methylation by diazomethane.
