# Supplementary material for: In Vivo Assay Reveals Microbial OleA Thiolases Initiating Hydrocarbon and β-Lactone Biosynthesis
Source: mBio. 2020 Mar 10;11(2):e00111-20. doi: 10.1128/mBio.00111-20 (PMC7064751; doi:10.1128/mBio.00111-20)
Supplement: TABLE S3 [file mBio.00111-20-st003.pdf]

| Source organism | Enzyme activity |
|---|---|
| *Kytococcus sedentarius* | 2.88 |
| *Mobilicoccus massiliensis* | 2.58 |
| *Granulosicoccus antarcticus* | 2.54 |
| *Xanthomonas campestris* | 2.44 |
| *Luteimonas tolerans* | 2.25 |
| *Thermomonas haemolytica* | 2.18 |
| *Xanthomonas translucens* | 2.17 |
| *Chromatocurvus halotolerans* | 2.17 |
| *Arenimonas oryziterrae* | 2.14 |
| *Kocuria flava* | 2.09 |
| *Pseudoxanthomonas* | 2.08 |
| *Intrasporangiaceae bacterium URHB0013* | 1.95 |
| *Actinoplanes atraurantiacus* | 1.93 |
| *Arthrobacter globiformis* | 1.87 |
| *Silanimonas lenta* | 1.78 |
| *Auraticoccus monumenti* | 1.75 |
| *Micromonospora peucetia* | 1.73 |
| *Mycobacterium obuense* | 1.73 |
| *Dermabacter hominis* | 1.71 |
| *Kocuria varians* | 1.7 |
| *Halobacteriovorax marinus* | 1.7 |
| *Sediminihabitans luteus* | 1.67 |
| *Leifsonia sp. Leaf325* | 1.64 |
| *Humibacillus sp. DSM 29435* | 1.63 |
| *Dermatophilus congolensis* | 1.63 |
| *Brachybacterium alimentarium* | 1.63 |

**Table S3.** Average enzyme activity ($\log_{10}$ of nmol pNP produced over the course of one hour by a *E. coli* BL21 culture with an OD of 1.0) for 73 OleAs.
