# Supplementary material for: In Vivo Assay Reveals Microbial OleA Thiolases Initiating Hydrocarbon and β-Lactone Biosynthesis
Source: mBio. 2020 Mar 10;11(2):e00111-20. doi: 10.1128/mBio.00111-20 (PMC7064751; doi:10.1128/mBio.00111-20)
Supplement: FIG S2 [file mBio.00111-20-sf002.pdf]

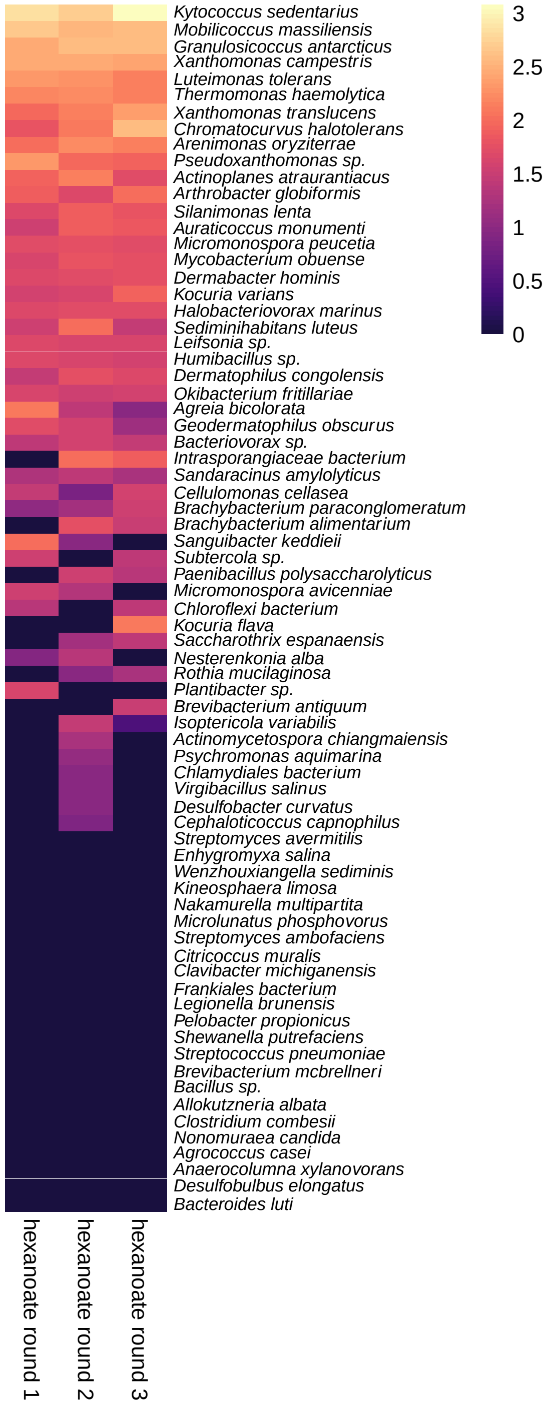

Figure S2. Heatmap of relative enzyme activity ($\log_{10}$ of nmol pNP produced over the course of one hour by a *E. coli* BL21 culture with an OD of 1.0) of 73 OleA enzymes across three different replicates. Enzyme activity ranges from high activity (yellow) to no activity (dark purple). Results reveal general reproducibility of this whole-cell method for enzymes with high activity and lower reproducibility for enzymes with weak activity.
