# Supplementary material for: In Vivo Assay Reveals Microbial OleA Thiolases Initiating Hydrocarbon and β-Lactone Biosynthesis
Source: mBio. 2020 Mar 10;11(2):e00111-20. doi: 10.1128/mBio.00111-20 (PMC7064751; doi:10.1128/mBio.00111-20)
Supplement: FIG S3 [file mBio.00111-20-sf003.pdf]

- *Kytococcus sedentarius*
- *Mobilicoccus massiliensis*
- *Granulosicoccus antarcticus*
- *Xanthomonas campestris*
- *Luteimonas tolerans*
- *Pseudoxanthomonas*
- *Thermomonas haemolytica*
- *Arenimonas oryziterrae*
- *Xanthomonas translucens*
- *Actinoplanes atraurantiacus*
- *Arthrobacter globiformis*
- *Chromatocurvus halotolerans*
- *Micromonospora peucetia*
- *Humibacillus sp. DSM 29435*
- *Halobacteriovorax marinus*
- *Leifsonia sp. Leaf325*
- *Silanimonas lenta*
- *Dermabacter hominis*
- *Mycobacterium obuense*
- *Kocuria varians*
- *Sediminihabitans luteus*
- *Auraticoccus monumenti*
- *Dermatophilus congolensis*

Biological replicate 1

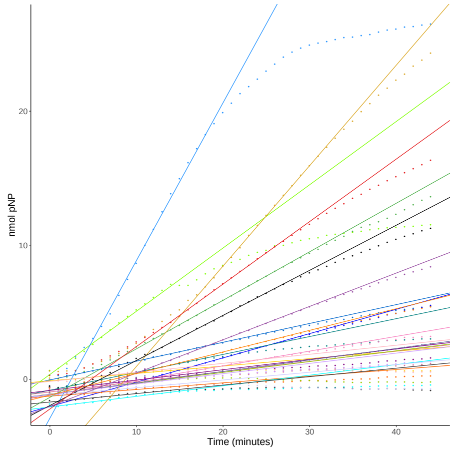

Source organism	Enzyme activity
Kytococcus sedentarius	2.85
Mobilicoccus massiliensis	2.65
Granulosicoccus antarcticus	2.45
Xanthomonas campestris	2.45
Luteimonas tolerans	2.34
Pseudoxanthomonas	2.3
Thermomonas haemolytica	2.17
Arenimonas oryziterrae	2.04
Xanthomonas translucens	1.99
Actinoplanes atraurantiacus	1.93
Arthrobacter globiformis	1.9
Chromatocurvus halotolerans	1.82
Micromonospora peucetia	1.71
Humibacillus sp. DSM 29435	1.69
Halobacteriovorax marinus	1.68
Leifsonia sp. Leaf325	1.67
Silanimonas lenta	1.66
Dermabacter hominis	1.66
Mycobacterium obuense	1.61
Kocuria varians	1.58
Sediminihabitans luteus	1.54
Auraticoccus monumenti	1.52
Dermatophilus congolensis	1.47

Biological replicate 2

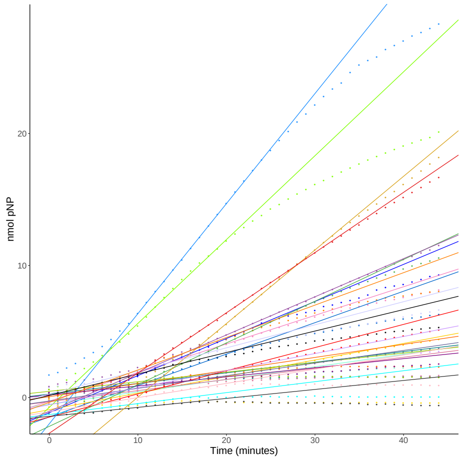

Source organism	Enzyme activity
Kytococcus sedentarius	2.7
Granulosicoccus antarcticus	2.58
Mobilicoccus massiliensis	2.52
Xanthomonas campestris	2.44
Luteimonas tolerans	2.28
Thermomonas haemolytica	2.24
Arenimonas oryziterrae	2.23
Xanthomonas translucens	2.17
Actinoplanes atraurantiacus	2.15
Chromatocurvus halotolerans	2.11
Intrasporangiaceae bacterium URHB0013	2.02
Sediminihabitans luteus	2.02
Pseudoxanthomonas	1.99
Silanimonas lenta	1.89
Auraticoccus monumenti	1.88
Mycobacterium obuense	1.81
Dermatophilus congolensis	1.77
Micromonospora peucetia	1.76
Brachybacterium alimentarium	1.76
Dermabacter hominis	1.7
Halobacteriovorax marinus	1.7
Arthrobacter globiformis	1.68
Humibacillus sp. DSM 29435	1.64
Leifsonia sp. Leaf325	1.62
Kocuria varians	1.61

Biological replicate 3

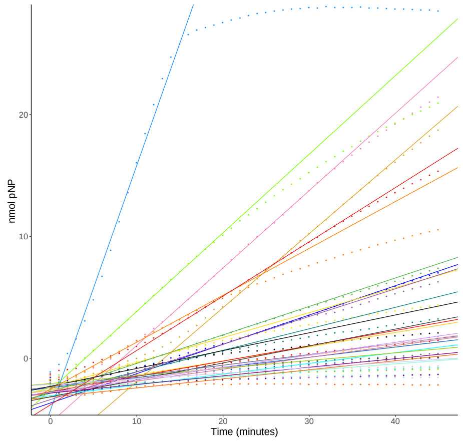

Source organism	Enzyme activity
Kytococcus sedentarius	3.08
Granulosicoccus antarcticus	2.59
Chromatocurvus halotolerans	2.58
Mobilicoccus massiliensis	2.56
Xanthomonas campestris	2.43
Xanthomonas translucens	2.36
Arenimonas oryziterrae	2.16
Luteimonas tolerans	2.15
Thermomonas haemolytica	2.13
Kocuria flava	2.09
Arthrobacter globiformis	2.02
Pseudoxanthomonas	1.94
Kocuria varians	1.92
Intrasporangiaceae bacterium URHB0013	1.88
Auraticoccus monumenti	1.85
Silanimonas lenta	1.79
Dermabacter hominis	1.77
Mycobacterium obuense	1.76
Halobacteriovorax marinus	1.72
Micromonospora peucetia	1.72
Actinoplanes atraurantiacus	1.7
Dermatophilus congolensis	1.65
Leifsonia sp. Leaf325	1.63
Humibacillus sp. DSM 29435	1.58
Brachybacterium alimentarium	1.51
Sediminihabitans luteus	1.44

Figure S3. Graph of active OleA enzymes in whole cell *p*-NP assays across all 3 replicates, along with a reference line for calculated slope. Active organisms for each replicate are listed besides the table as well as rate of enzyme activity in nmol p-NP / OD 1.0 / hour.
