# Supplementary material for: In Vivo Assay Reveals Microbial OleA Thiolases Initiating Hydrocarbon and β-Lactone Biosynthesis
Source: mBio. 2020 Mar 10;11(2):e00111-20. doi: 10.1128/mBio.00111-20 (PMC7064751; doi:10.1128/mBio.00111-20)
Supplement: FIG S4 [file mBio.00111-20-sf004.pdf]

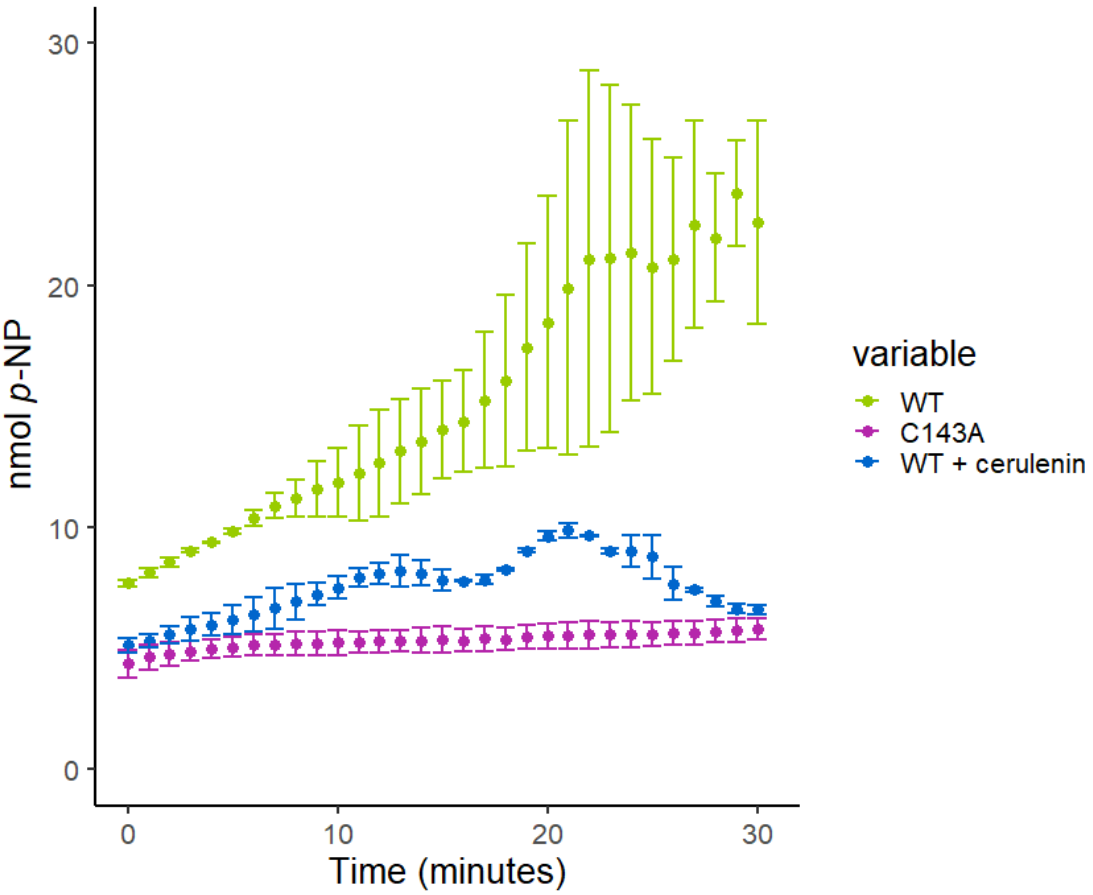

Figure S4. 4 µg of OleA WT (green) and OleA C143A (magenta) and WT with 25 µM cerulenin (blue) were assayed with 200 µM *p*-nitrophenyl dodecanoate in 50 mM Tris HCl pH 8 at 37 C. Cerulenin was added prior to the assay and the plate was allowed to incubate for 10 minutes at 37 C.
