# Supplementary material for: In Vivo Assay Reveals Microbial OleA Thiolases Initiating Hydrocarbon and β-Lactone Biosynthesis
Source: mBio. 2020 Mar 10;11(2):e00111-20. doi: 10.1128/mBio.00111-20 (PMC7064751; doi:10.1128/mBio.00111-20)
Supplement: FIG S5 [file mBio.00111-20-sf005.pdf]

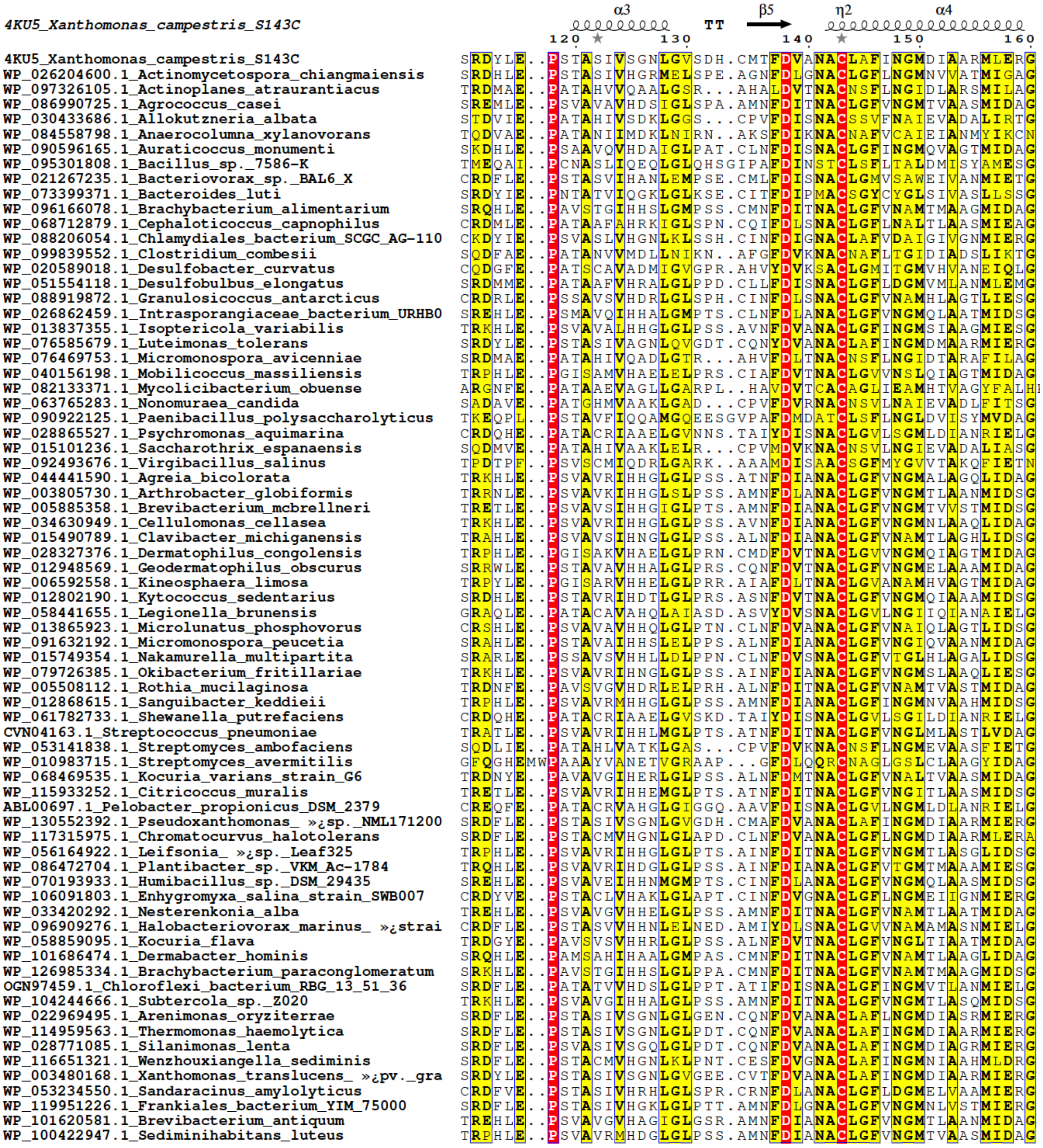

Figure S5. Structure-based multiple sequence alignment of OleA proteins screened in this study. The numbering and structural elements depicted are based on the X-ray structure of the *X. campestris* OleA. Glutamate 117 is highly conserved, proline 118 and cysteine 143 are completely conserved.
