# Supplementary material for: In Vivo Assay Reveals Microbial OleA Thiolases Initiating Hydrocarbon and β-Lactone Biosynthesis
Source: mBio. 2020 Mar 10;11(2):e00111-20. doi: 10.1128/mBio.00111-20 (PMC7064751; doi:10.1128/mBio.00111-20)
Supplement: FIG S6 [file mBio.00111-20-sf006.pdf]

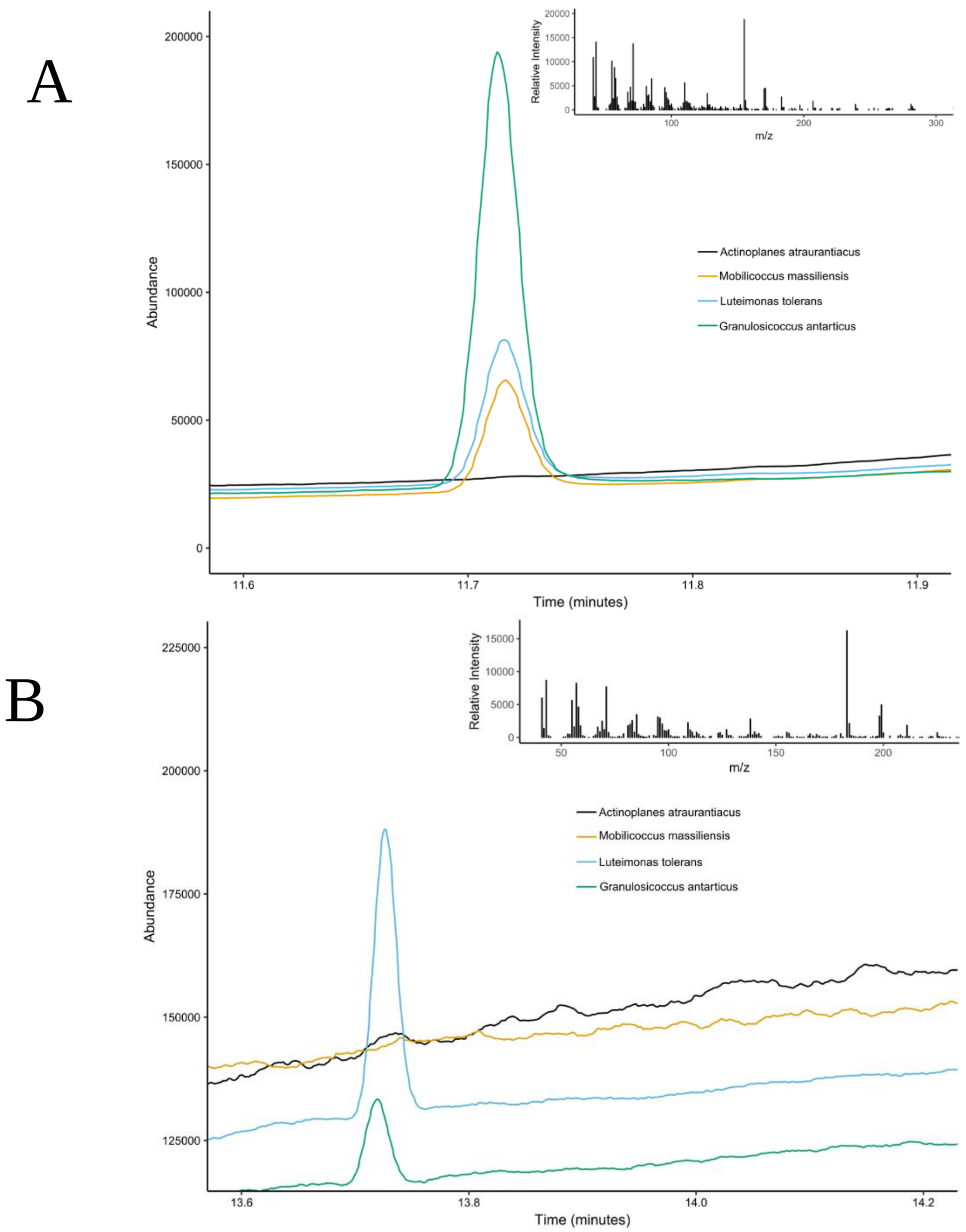

Figure S6. A.) GC-FID/MS split chromatogram of an extract from reaction of OleA proteins from different sources with decanoyl-CoA showing 10-nonadecanone, the stable decarboxylated product of the Claisen condensation that can be detected by GC. Black is *A. atraurantiacus*, orange is *M. massiliensis*, blue is *L. tolerans*, green is *G. antarticus*. Inset shows the MS spectrum of the peak (from the representative *Luteimonas tolerans* OleA). All mass spectra from reactions showing a peak gave the characteristic fragments for 10-noadecanone. **B.)** GC-FID/MS split chromatogram of an extract from reaction of OleA proteins from different sources with lauryl-CoA showing 12-tricosanone, the stable decarboxylated product of the Claisen condensation that can be detected by GC. Black is *A. atraurantiacus*, orange is *M. massiliensis*, blue is *L. tolerans*, green is *G. antarticus*. Inset shows the MS spectrum of the eluted standard peak. All mass spectra from reactions showing a peak gave the characteristic fragments for 12-tricosanone.
