# Supplementary material for: In Vivo Assay Reveals Microbial OleA Thiolases Initiating Hydrocarbon and β-Lactone Biosynthesis
Source: mBio. 2020 Mar 10;11(2):e00111-20. doi: 10.1128/mBio.00111-20 (PMC7064751; doi:10.1128/mBio.00111-20)
Supplement: FIG S7 [file mBio.00111-20-sf007.pdf]

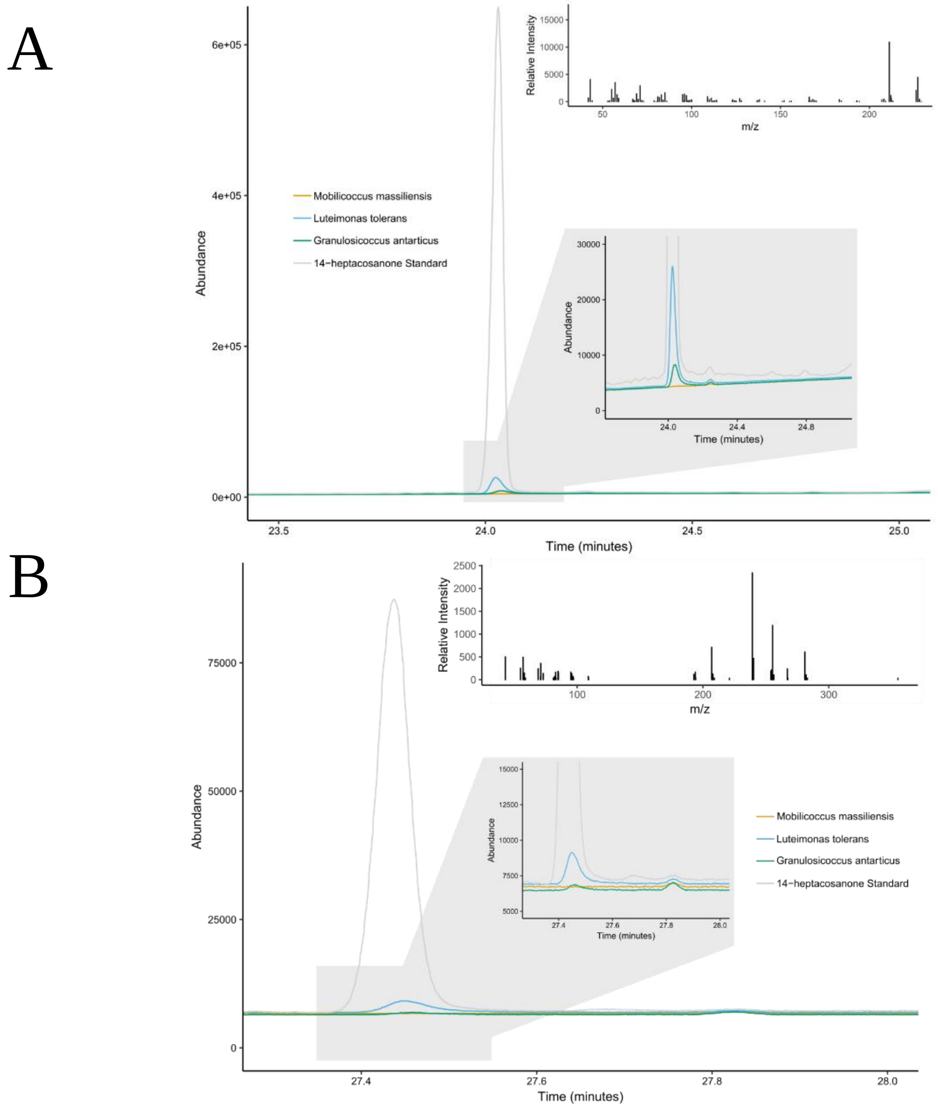

Figure S8. A.) GC-FID/MS split chromatogram of an extract from reaction of OleA proteins from different sources with myristoyl-CoA showing 14-heptacosanone, the stable decarboxylated product of the Claisen condensation that can be detected by GC. Orange is *M. massiliensis*, blue is *L. toleran*, green is *G. antarticus*, grey is the chemical standard. *A. atraurantiacus* was not included due to inactivity and insolubility. Inset shows the MS spectrum of the eluted standard peak. All mass spectra from reactions showing a peak gave the characteristic fragments for 14-heptacosanone. **B.)** GC-FID/MS split chromatogram of an extract from reaction of OleA proteins from different sources with palmitoyl-CoA showing 16-hentriacontanone, the stable decarboxylated product of the Claisen condensation that can be detected by GC. Orange is *M. massiliensis*, blue is *L. tolerans*, green is *G. antarcticus*, grey is the chemical standard. *A. atraurantiacus* was not included due to inactivity and insolubility. Inset shows the MS spectrum of the eluted standard peak. All mass spectra from reactions showing a peak gave the characteristic fragments for 16-hentriacontanone.
